# Inhibition of SMYD2 Sensitized Cisplatin to Resistant Cells in NSCLC Through Activating p53 Pathway

**DOI:** 10.3389/fonc.2019.00306

**Published:** 2019-04-26

**Authors:** Lei Shang, Minjie Wei

**Affiliations:** ^1^School of Pharmacy, China Medical University, Shenyang, China; ^2^Shenyang Medical College, Shenyang, China

**Keywords:** SMYD2, cisplatin resistance, lung cancer, p53, apoptosis

## Abstract

The protein lysine methyltransferase SMYD2 has recently emerged as a new enzyme modulate gene transcription or signaling pathways, and involved into tumor progression. However, the role of SMYD2 in drug resistant is still not known. Here, we found that inhibition of SMYD2 by specific inhibitor could enhance the cell sensitivity to cisplatin (CDDP), but not paclitaxel, NVB, and VCR in non-small cell lung cancer (NSCLC). Further study showed that SMYD2 and its substrates were overexpressed in NSCLC resistant cells, and the inhibition of SMYD2 or knockdown by specific siRNA could reverse the cell resistance to cisplatin treatment in NSCLC/CDDP cells. In addition, our data indicated that the inhibition or knockdown SMYD2 inhibit tumor sphere formation and reduce cell migration in NSCLC/CDDP cells, but not in NSCLC parental cells. Mechanistically, inhibition of SMYD2 could enhance p53 pathway activity and induce cell apoptosis through regulating its target genes, including p21, GADD45, and Bax. On the contrary, the sensitivity of cells to cisplatin was decreased after knockdown p53 or in p53 deletion NSCLC cells. The synergistically action was further confirmed by *in vivo* experiments. Taken together, our results demonstrate SMYD2 is involved into cisplatin resistance through regulating p53 pathway, and might become a promising therapeutic target for cisplatin resistance in NSCLC.

## Introduction

The incidence and mortality of lung cancer ranks at the NO.1 among all kinds of cancer (1). Non-small cell lung cancer (NSCLC) accounts for about 85% of lung cancer (1, 2). The surgery, radiotherapy, chemotherapy, molecular targeting therapy, and immunotherapy are possible choice for NSCLC treatment (2). However, most of NSCLCs are found at advanced stage, so drug-based therapy, mainly including chemotherapy, is considered as the most important approach to treat NSCLCs (3).

The platinum-based chemotherapy, such as cisplatin plus paclitaxel, cisplatin plus NVB, and cisplatin plus VCR, is the first-line treatment approach in NSCLCs (2, 3). However, drug resistance will be inevitable happened after treatment for 1–2 years, which limit the application of chemotherapeutic agents (4, 5). To solve this problem, we should first understand the resistant mechanisms for chemotherapy in NSCLCs. In fact, many previous studies have shown that the upregulation of efflux protein, the mutation of drug target, the activation of by-pass oncogenic pathway, and the accumulation of phenotype change cells contributed to the resistance of chemotherapeutic agents in NSCLCs (6, 7). However, there is still unknown for a large part of NSCLC resistant patients.

SMYD2 was identified as protein methyltransferase which adds methyl-group to its histone and non-histone substrates and epigenetically regulates their function (8, 9). Recently, SMYD2 was observed to involve into the upset and progression of various tumors including leukemia, breast cancer, teratocarcinoma, gastric cancer, and head and neck cancer (10–14). Mechanistically, SMYD2 was found to prompt cell proliferation, block apoptosis, and enhance cell migration and invasion through regulating its substrates methylation status, such as p53 and histone4 (13–15). However, whether this enzyme is involved into drug resistance is still not known.

Here, NSCLC was used to as an example to investigate the role of SMYD2 in chemotherapeutic resistance. Our data showed that SMYD2 was involved into cisplatin resistance, but not paclitaxel, NVB, and VCR. Further study indicated that SMYD2 expression and its activity were increasing in cisplatin resistant NSCLC cells. Mechanistically, SMYD2 prompt cell migration, increase the tumor sphere and block apoptosis, which is dependent on the methylation of p53^K370^. The inhibition or knockdown of SMYD2 model would result in the increasing of sensitivity to cisplatin *in vitro* and *in vivo*. Our results not only elucidate the role of SMYD2 in cisplatin resistance and provide a potential method to reverse cisplatin resistance in NSCLC.

## Materials and Methods

### Cell Lines, Cell Culture, and Treatment

A549 (p53 wide type, KRAS mutation), NCI-H460 (p53 wide type, KRAS mutation), and NCI-H1299 (p53 deletion, KRAS wide type) human lung adenocarcinoma cell lines were purchased from the American Type Culture Collection (ATCC; Manassas, VA, USA). These cancer cells were routinely cultured in RPMI-1640 medium (Gibco, Grand Island, NY, USA) supplemented with 10% fetal bovine serum (FBS; Gibco) and were maintained at 37°C in a humidified incubator with 5% CO_2_. The cells were treated with Cisplatin (J&K Scientific Ltd, Beijing, China) at increasing concentrations (ranging from 0.5 to 4 μM) for 3 months.

### Compounds and Reagents

BAY-498(SMYD2 inhibitor), AZ505(SMYD2 inhibitor), Cisplatin(CDDP), Vinorelbine(NVB), Paclitaxel (Taxol), and Vincristine sulfate(VCR) was obtained from MedChem Express (Princeton, NJ, USA). The primary antibodies against SMYD2, p53, Cleaved-PARP, and β-actin were obtained from Cell Signaling Technology (Danvers, MA, USA), and the primary antibodies against p53^K370Me^ was purchased from Immunoway Technology (Plano, TX, USA). The pcDNA3-p53 vector was obtained from Addgene.

### Cell Viability Assay

*In vitro* cell viability was determined using the MTT assay. Cells (1 × 10^5^ cells/ml) were seeded in 96-well culture plates. After incubating overnight, the cells were treated with various concentrations of the appropriate agents for 48 h, after which 10 μl of MTT solution (2.5 mg/ml in PBS) was added to each well, and the plates were incubated for an additional 4 h at 37°C. After the samples were centrifuged (2,500 rpm, 10 min), the medium supplemented with MTT was aspirated, and then 100 μl of DMSO was added to each well. The optical density of each well was measured at 570 nm with a Biotek Synergy^TM^ HT Reader (BioTek Instruments, Winooski, VT, USA).

### Western Blot Analysis

Western blotting was performed as previously described (14). Briefly, equal amounts of total protein extracts from cultured cells or tissues were fractionated by 10–15% SDS-PAGE before being electrically transferred onto polyvinylidene difluoride (PVDF) membranes, which were sequentially incubated with mouse or rabbit primary antibodies and horseradish peroxidase (HRP)-conjugated secondary antibodies designed to detect the proteins of interest. The indicated secondary antibodies were subsequently reacted with ECL detection reagents (Pierce, Thermo Fisher Scientific, Waltham, MA, USA) and then incubated in a dark room. The relative expression levels of the indicated proteins were normalized to those of β-actin.

### Flow Cytometry Analysis

Analyses for apoptosis were conducted with an Annexin V-FITC Apoptosis Detection Kit (BioVision, Mountain View, CA, USA). Cells (1 × 10^6^) were exposed to various inhibitors for 48 h. They were collected by centrifugation and resuspended in 500 μL of 1 × binding buffer. Annexin V-fluorescein isothiocyanate (FITC; 5 μL) and PI (5 μL) were added to the cells. After incubation at room temperature for 5 min in the dark, cells were analyzed by FACS using a flow cytometer (BD Biosciences, San Jose, CA, USA). Cells that stained Annexin V-FITC (apoptosis) were analyzed.

### siRNA-Mediated Gene Knockdown

*SMYD2* and *p53* knockdown was performed using specific siRNAs purchased from Santa Cruz Biotechnology (Santa Cruz Biotechnology, Santa Cruz, CA, USA). Scramble non-target siRNAs served as negative controls. siRNA was introduced into the indicated cell lines with Lipofectamine RNAiMAX reagent (Thermo Fisher Scientific), according to the manufacturer's instructions, and knockdown efficiency was assessed by western blotting.

### Transwell Migration Assay

NCI-H460/CDDP and its parental cell lines migration capacities were tested by Corning transwell assay, according to the manufacturer's instructions. Briefly, the indicated lung cancer cells were treated DMSO, BAY-598 (200 nM), Scramble siRNA, and SMYD2 siRNA (50 nM) for 48 h and then seeded in the upper chamber of the system at a density of 5 × 10^4^ cells/well in serum-free medium (100 μl). The wells in the lower chamber of the system were filled with complete medium. After incubating for 48 h, the cells remaining in the upper chamber were carefully removed with a cotton swab, and the cells that had migrated through the membrane and adhered to its lower surface were fixed with 100% methanol and stained with 0.2% crystal violet. The membrane was then photographed under a microscope, and the cells in five predetermined fields were counted at 200× magnification.

### Tumor Sphere Formation Assay

NCI-H460/CDDP and its parental cell lines were treated DMSO, BAY-598 (200 nM), Scramble siRNA, and SMYD2 siRNA (50 nM) for 48 h, after which single cells prepared by mechanical and enzymatic dissociation were seeded in 6-well ultra-low attachment plates (Corning, NY, USA) at a density of 1,000 cells/well in serum-free DMEM/F-12 medium supplemented with B27 (1×, Invitrogen, Thermo Fisher Scientific), 20 ng/ml human recombinant bFGF (PeproTech, Rocky Hill, NJ, USA), and 20 ng/ml EGF (PeproTech) for 10–14 days. The cells were then photographed under a microscope.

### Luciferase Reporter Gene Assays

NCI-H460/CDDP and its parental cells were plated in 96-well plates. Cells in 96-well plates were transfected with 2 ng pRL-tk (Promega) and 50 ng p53 reporter plasmid (Addgene) for 24 h with the lipofectamine 3000. Cells were treated with DMSO or BAY-598 at indicated concentrations for 24 h. Luciferase activities were evaluated with the Berthold LB960 system (Berthold, DE).

### Quantitative PCR Analysis

Total RNA was isolated using an RNeasy Mini Kit (Qiagen, Hilden, Germany), as described in the product insert, and then reverse transcribed with a RevertAid First Strand cDNA Synthesis Kit (Thermo Fisher Scientific). PCR was performed with iQ SYBR Green SuperMix (Bio-Red Laboratories, Hercules, CA, USA) and a CFX96 Real-Time PCR Detection System (Bio-Rad Laboratories). The following primers were used for the experiment: glyceraldehyde-3-phosphate dehydrogenase (*GAPDH*): reverse: 5′-CCCTCAACGACCACTTTGTCA-3′ and forward: 5′-TTCCTCTTGTGCTCTTGCTGG-3′; p21 forward: 5′-TGTACCCTTGTGCCTCGCTC-3′ and reverse: 5′- TGGAGAAGATCAGCCGGCGT-3′; Bax forward: 5′- TTTGCTTCAGGGTTTCATCC-3′ and reverse: 5′- CAGTTGAAGTTGCCGTCAGA-3′; and GADD45 forward: 5′-GGATGCCCTGGAGGAAGTGCT-3′ and reverse: 5′- GGCAGGATCCTTCCATTGAGATGAATGTG-3′.

### Xenografts in Mice

To assess the characteristics of chemotherapy-resistant tumors, we subcutaneously injected viable NCI-H460/CDDP cells (5 × 10^6^/100 μl PBS per mouse), as confirmed by trypan blue staining, into the right flank of 7–8 weeks-old male BALB/C mice. When the average tumor volume reached 100 mm^3^, the mice were randomly divided into the following four treatment groups: a control group (saline only, *n* = 6), a AZ505 group (40 mg/kg/qd, i.p.; *n* = 6), an CDDP group (4.0 mg/kg/3 day, i.p.; *n* = 6), and a combination treatment group (AZ505 plus CDDP). After 2 weeks, the mice were sacrificed, and the tumors were excised and stored at −80°C. These experiments were performed in strict accordance with the recommendations in the Guide for the Care and Use of Laboratory Animals of the National Institutes of Health, and the corresponding protocol was approved by the Animal Experimental Ethics Committee of Shenyang Medical College (Shenyang, Liaoning Province, China).

### Statistical Analysis

Differences between the indicated experimental groups were evaluated by one-way ANOVA or Turkey's *post hoc* test with the SPSS 11.5 software package for Windows (SPSS, Chicago, IL, USA). *P* < 0.05 were considered statistically significant (*P* < 0.05, two-tailed test).

## Results

### The Inhibition of SMYD2 Enhanced the Antigrowth Action of Cisplatin in NSCLC Cells

**Figure 1:**
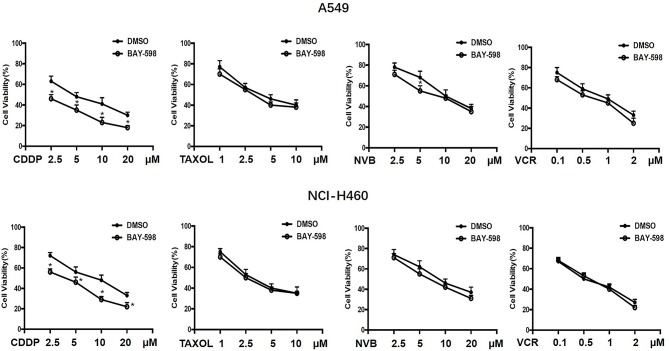
Effects of the combination of chemotherapeutic agents and SMYD2 inhibitor on cell growth in NSCLC cells. The growth of A549 and NCI-H460 cells treated with chemotherapeutic agents, including CDDP, Taxol, NVB, and VCR at different concentrations or combination with SMYD2 inhibitor BAY-598. Cell lines treated with DMSO were used as controls.

To explore the possible action of SMYD2 in chemotherapeutic agents in NSCLC, A549 and NCI-H460 cells were treated with various concentrations of the first-line chemotherapeutic agents, including CDDP, Taxol, NVB, and VCR, and combined treatment with SMYD2 inhibitor BAY-598 with non-cytotoxicity concentration (2 μM, cell viability>90%, Supplementary Figure 1A). As shown in Figure 1, single treatment with CDDP, Taxol, NVB, and VCR could inhibit cell growth at concentration-dependent manner in both cell lines. Addition of SMYD2 inhibitor had no effect on the cell viability when combined with Taxol and VCR, and owned a slightly enhanced inhibition when combined with NVB. Notably, the combination of BAY-598 and CDDP could significantly retard cell growth in both A549 and NCI-H460 cells (*P* < 0.05), suggesting SMYD2 inhibition might be involved into the cell sensitivity to CDDP but not Taxol, VCR, and NVB.

### The Expression and Function of SMYD2 in Cisplatin Resistant NSCLC Cells

**Figure 2:**
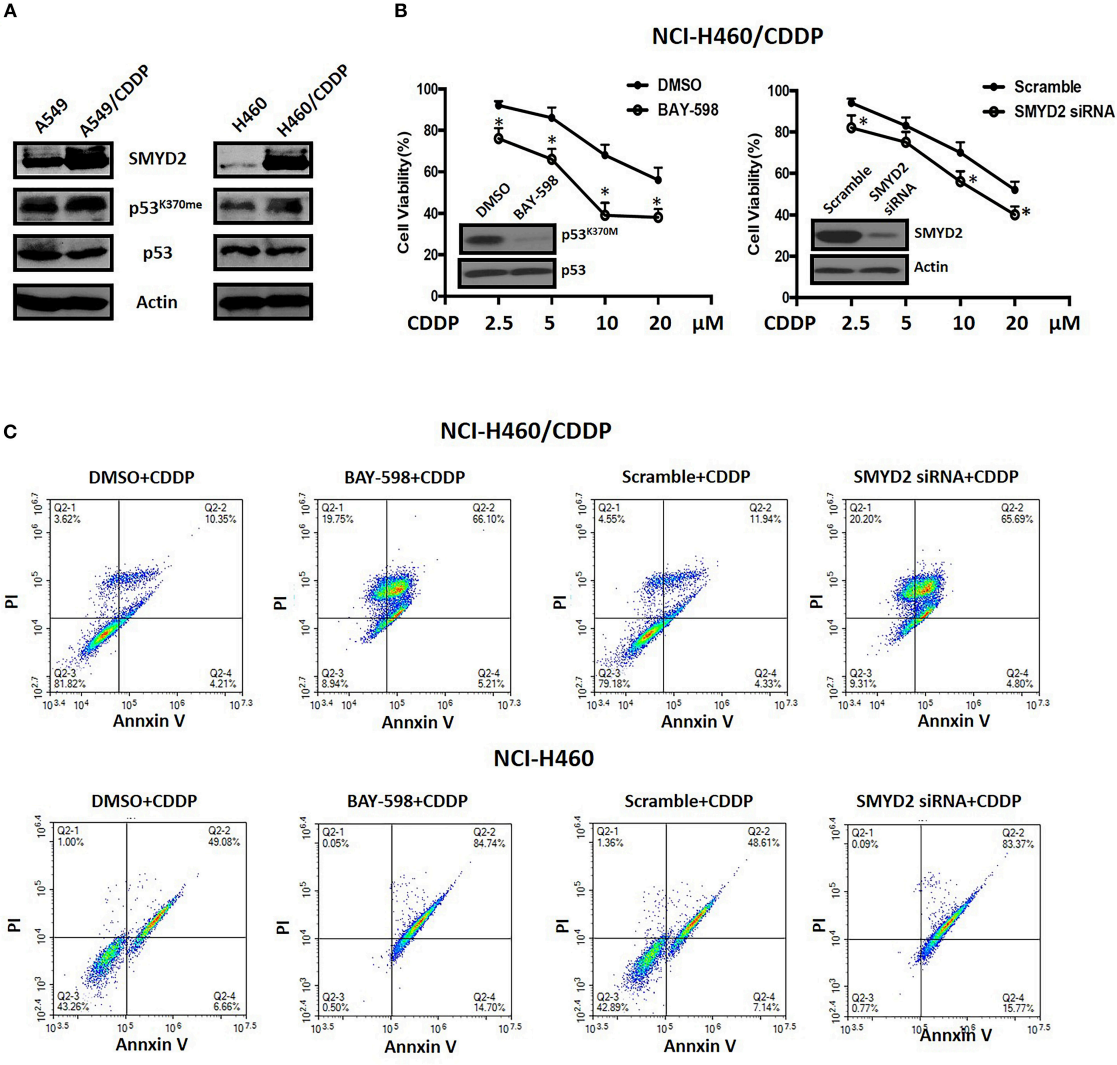
The expression level of SMYD2-related proteins and the effects of genetic or chemical manipulation of SMYD2 on the cell growth of CDDP-resistant and parental NSCLC cells. **(A)** SMYD2, p53, and p53^K370me^ expression levels were measured in CDDP resistant and parental NSCLC cell lines. β-actin was used as a loading control. **(B)** Cell viability was measured in BAY-598-treated or SMYD2-knockdown NCI-H460/CDDP cells treated with CDDP at different concentrations for 36 h. Scramble siRNA or DMSO was used as a control. The efficacy of genetic or chemical manipulation of SMYD2 was confirmed by Western blot in NCI-H460/CDDP cells. **(C)** Cell apoptosis was assessed using Annexin V/PI double staining in BAY-598-treated or SMYD2-knockdown CDDP resistant and parental NCI-H460 cells after treated with CDDP at 10 μM for 48 h. ^*^P < 0.05, compared to corresponding control cells.

To clarify the role and function of SMYD2 in CDDP sensitivity of NSCLC cells, we established A549 and NCI-H460 CDDP resistant cell lines. First, we detected the expression level of SMYD2 in parental cell lines and resistant cell lines. Western blot data indicated that SMYD2 was increased in both resistant cell lines as compared to parental cell lines. In consistent with the SMYD2 upregulation in resistant cell lines, the non-histone substrate of SMYD2, p53^K370me^, was also increased in resistant cell lines. The above data demonstrated that the expression and activity of SMYD2 were increased in CDDP resistant cells. Next, to further elucidate the role of SMYD2 in CDDP resistance, we measured the cell viability of NCI-H460/CDDP cells to CDDP after suppression of SMYD2 by specific inhibitor and siRNA. Our data showed, whether inhibition by SMYD2 inhibitor BAY-598 or knockdown by specific siRNA, the cell sensitivity to CDDP would be significantly increased as compared to DMSO or Scramble treated groups (*P* < 0.05). The above data was confirmed by flow cytometry experiments. Treatment with BAY-598 at non-cytotoxic concentration would prompt the apoptosis induced action of CDDP in NCI-H460/CDDP cells. Similarly, knockdown SMYD2 also resulted in the increase of cell apoptosis in CDDP treated NCI-H460/CDDP cells when compared to scramble treated cells. Notably, although the addition of SMYD2 inhibitor or knockdown of SMYD2 could enhance the induction of apoptosis by CDDP in NCI-H460 cells, the level was decreased as compared with resistant cells (Figure 2C). The above data indicated that SMYD2 play an important role in CDDP resistance of NSCLC cells.

### Inhibition of SMYD2 Reversed Malignant Phenotype of Cisplatin Resistant NSCLC Cells

To further elucidate the role of SMYD2 in CDDP resistance of NSCLC cells, we next assessed the effect of inhibition or knockdown of SMYD2 on cell migration and tumor sphere formation, which are considered as the crucial characteristics of CDDP resistant NSCLC cells (16, 17). Our results showed that cell migration number of NCI-H460/CDDP cells was significant decreased after treated with SMYD2 inhibitor or SMYD2 siRNA as compared to DMSO and Scramble siRNA control, respectively. Furthermore, tumor sphere number of NCI-H460/CDDP cells was also obviously reduced by SMYD2 inhibitor and SMYD2 siRNA. It should be noted that whether addition of BAY-598 or specific siRNA could not significantly affect cell migration number and tumor sphere ability in NCI-H460 cells (Supplementary Figures 1B–D). The above results demonstrated that SMYD2 was also involved into the formation of malignant phenotype in CDDP resistant NSCLC cells.

### SMYD2 Mediated Cisplatin Resistance Dependent on p53 Regulation in NSCLC Cells

In view of the crucial role of p53 and its epigenetic regulation by SMYD2 (18), we next explore possible role of p53 in SMYD2 mediated CDDP resistance. As shown in Figure 4A, knockdown p53 by specific siRNA contributed to the decrease of cell sensitivity to CDDP in NCI-H460/CDDP cells, which owned wide type p53 expression. In addition, the restore of p53 in NCI-H1299 cells (p53 deletion) could lead to the increase of cell sensitivity to CDDP. The above data indicates that the status and expression level of p53 will affect the cell sensitivity of NSCLC cells to CDDP.

**Figure 3:**
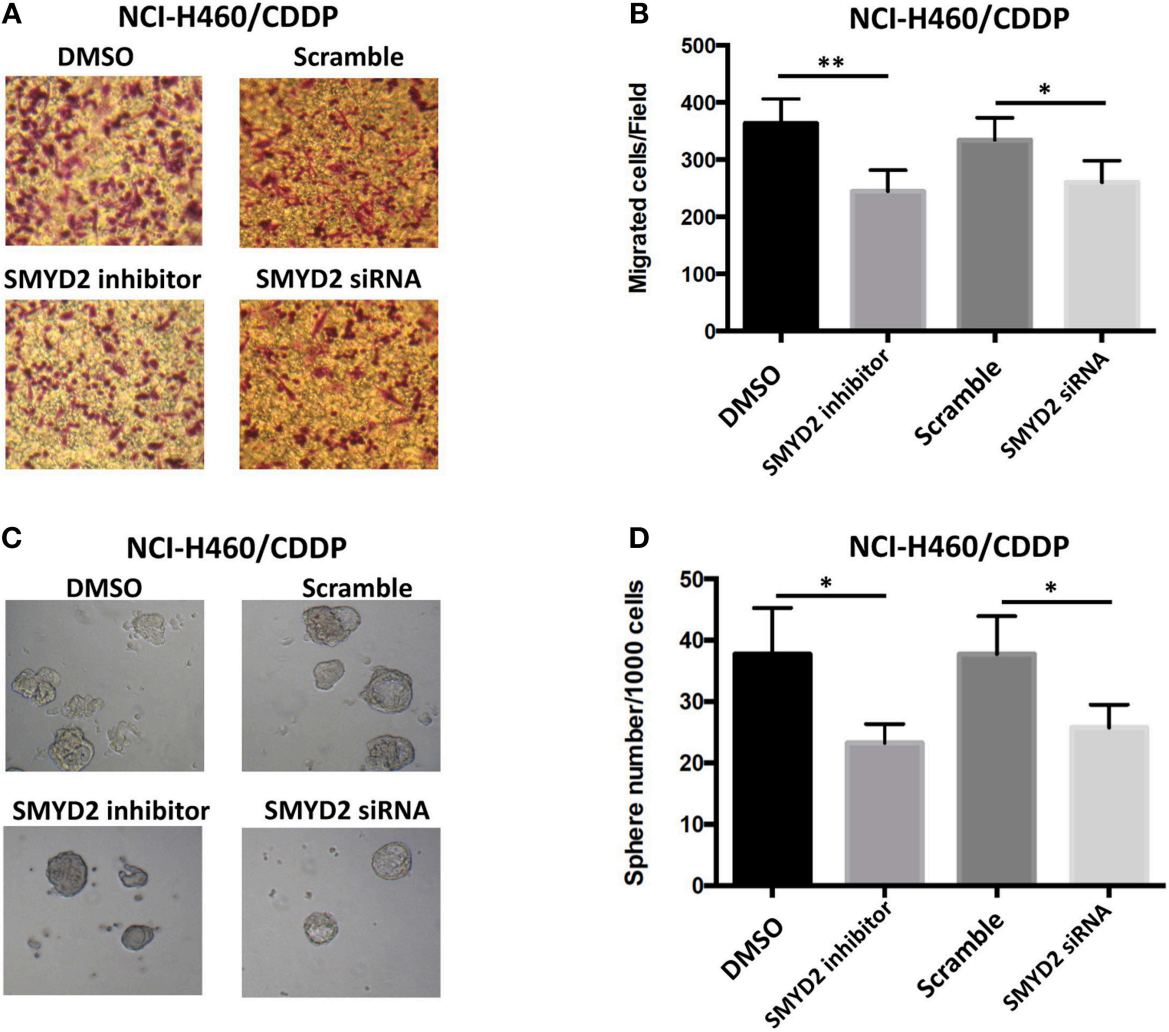
Effects of genetic or chemical manipulation of SMYD2 on the biological characteristics of CDDP-resistant NSCLC cells. **(A,B)** Cell migration was measured in NCI-H460/CDDP cells treated with 2 μM BAY-598 or 50 nM SMYD2 siRNA. Scramble siRNA or DMSO was used as a control. **(C,D)** Tumor sphere was counted in NCI-H460/CDDP cells treated with 2 μM BAY-598 or 50 nM SMYD2 siRNA. Scramble siRNA or DMSO was used as a control. (Scale bars, 100 μm) ^*^P < 0.05, ^**^P < 0.001, compared to corresponding control cells.

**Figure 4:**
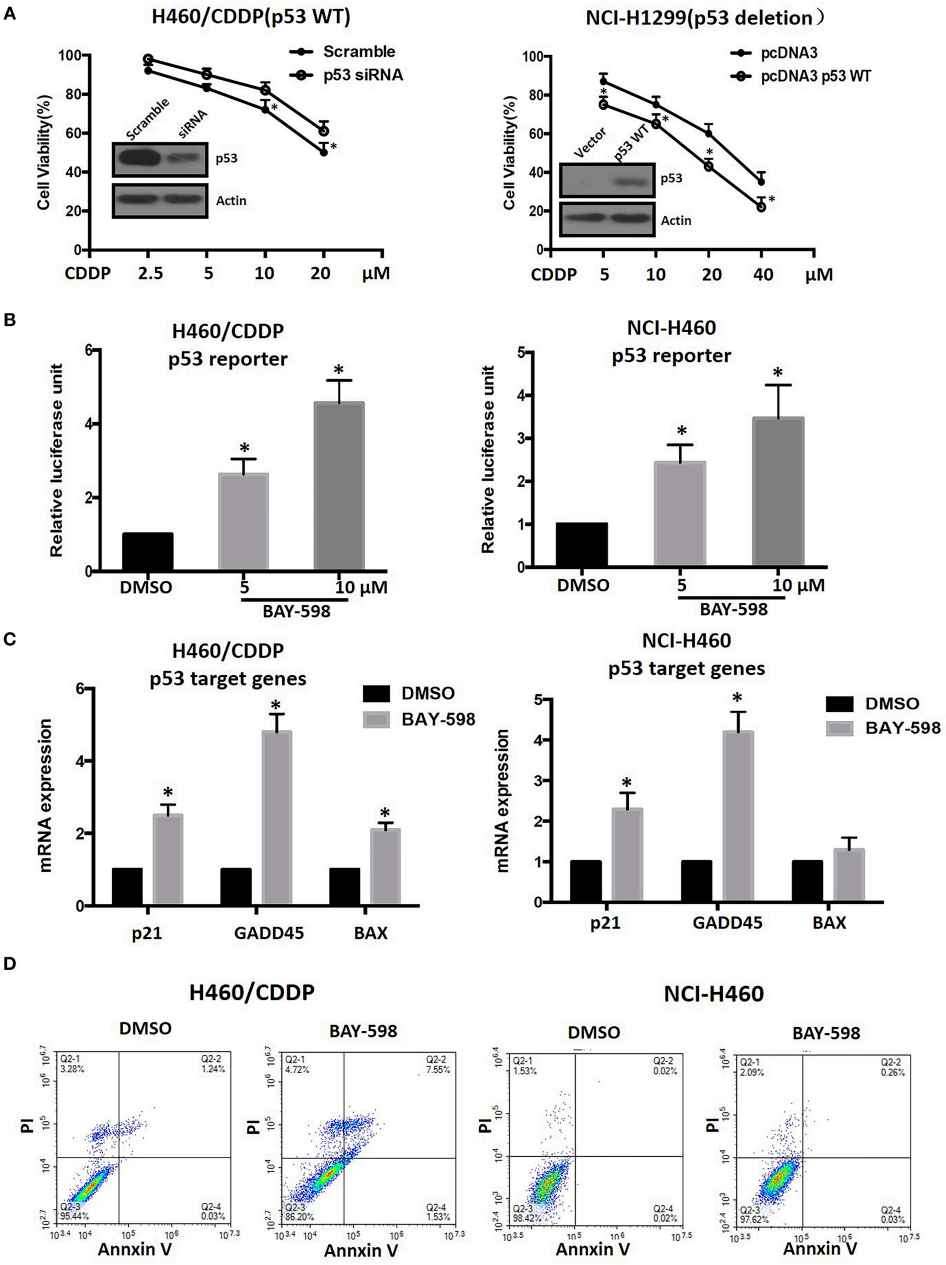
Epigenetic regulation of p53 and its role in CDDP resistance in NSCLC. **(A)** Cell viability in NCI-H460/CDDP (p53 wide type) and NCI-H1299(p53 deletion) cells, with p53 gene manipulation, which were treated with CDDP at different concentrations for 48 h. Scramble siRNA or mock vector was used as a control. The p53 knock-down or restoration efficacy was confirmed by Western Blot. **(B)** The p53 reporter activity was measured in CDDP resistant and parental NCI-H460 cells after treated with BAY-598. The relative luciferase unit was calculated by Luciferase/Renilla and DMSO was considered as 100%. **(C)** The mRNA expression levels of p21, GADD45, and Bax were assessed by real-time RT-PCR in CDDP resistant and parental NCI-H460 cells treated with 10 μM BAY-598. GAPDH was used as a control. **(D)** Cell apoptosis was assessed using Annexin V/PI double staining in CDDP resistant and parental NCI-H460 cells which were treated with BAY-598 at 10 μM concentrations for 48 h. ^*^P < 0.05, compared to corresponding control cells.

In order to explore the effect of SMYD2 on p53 activity, we detected the transcriptional regulation activity of p53 by luciferase assay after treated with BAY-598 in NCI-H460/CDDP and its parental cells. The results showed that BAY-598 could concentration-dependently enhance p53 reporter activity in NCI-H460/CDDP cells (Figure 4B). In consistent with reporter assay, BAY-598 treatment also significantly resulted in the upregulation in mRNA level of p53 targeting genes, including p21, GADD45, and Bax (Figure 4C), in NCI-H460/CDDP cells. In consistent with resistant cell lines, BAY-598 also could increase p53 reporter activity, p21 and GADD45 expressions in NCI-H460 cells to some extent (Figure 4B). On the contrary, BAY-598 treatment could not induce the BAX expression in NCI-H460 cells, suggesting the role of SMYD2 in BAX regulation is different in parental and resistant cells (Figure 4C). Furthermore, we also detected the cell apoptosis status of NCI-H460/CDDP and NCI-H460 cells after treated with BAY-598. Our results indicated BAY-598 at 10 μM could induce cell apoptosis in NCI-H460/CDDP cells, but not in NCI-H460 cells (Figure 4D), which confirmed the regulation action of Bax, a pro-apoptosis gene, by SMYD2. Taken together, our data suggested that the SMYD2 mediated CDDP resistance through epigenetic regulation of p53.

### Inhibition of SMYD2 Sensitized Cisplatin Through Epigenetic Regulation of p53 *in vivo*

**Figure 5:**
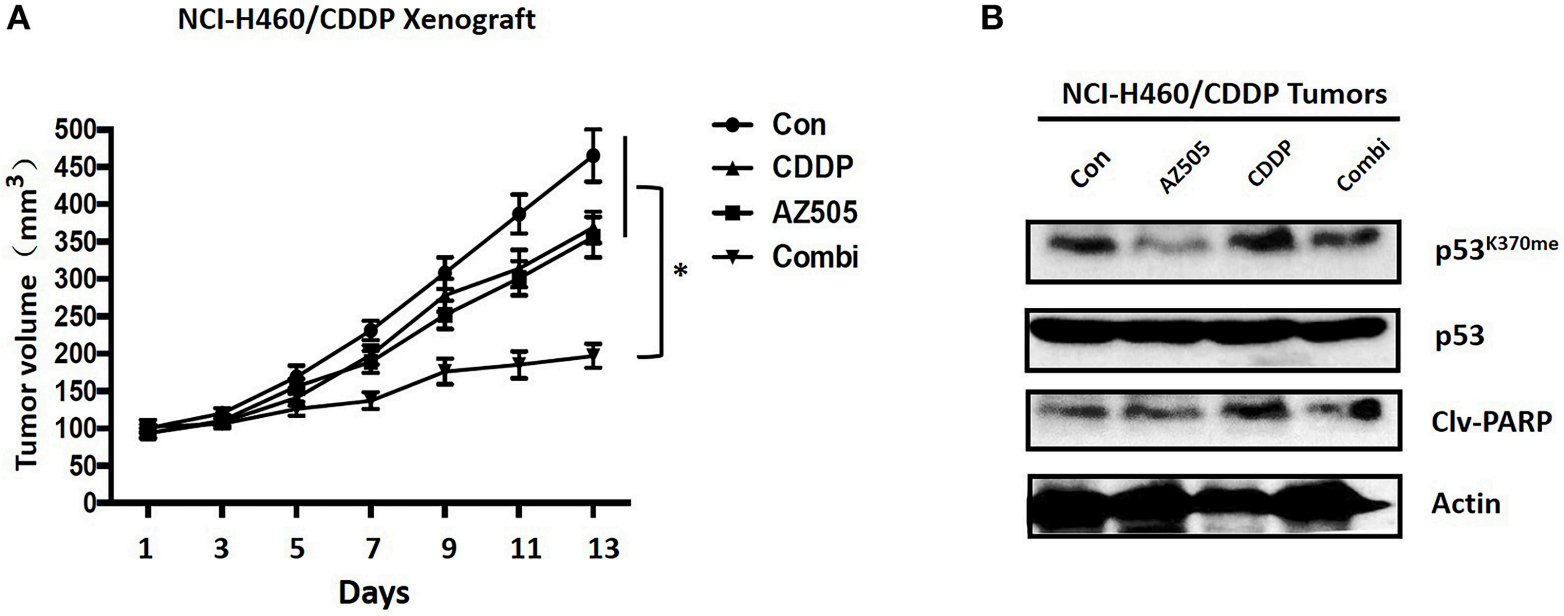
Effects of SMYD2 inhibition and/or CDDP on tumor growth in an CDDP-resistant xenograft model. **(A)** Tumor volume was measured in NCI-H460/CDDP xenografts treated with AZ505, CDDP, or the combination of AZ505 and CDDP. **(B)** The p53 and p53^K370me^, and cleaved PARP(clv-PARP) expression levels were measured in NCI-H460/CDDP xenograft tumor tissues. β-actin was used as a loading control. ^*^P < 0.05, combined treatment group compared to single treatment group and vehicle control.

To clarify the therapeutic meaning of the above finding, we assessed anti-tumor effect of the combination of SMYD inhibitor and CDDP in NCI-H1299/CDDP xenograft mice. As shown in Figure 5A, single treatment with CDDP has no significant effect on tumor growth, indicating the resistant phenotype of NCI-H1299/CDDP xenograft mice. Similar to CDDP single treatment, single treatment with AZ505, an *in vivo* available SMYD2 inhibitor, only displayed a slightly inhibition on tumor growth. Interestingly, the combination of AZ505 and CDDP could obviously inhibit tumor growth of NCI-H1299/CDDP xenograft mice when compared to vehicle control and single treatment group. In addition, we didn't find the body weight loss in the combination treated group (data not shown), suggesting the combination has no effect on gross toxicity. Next, we further explored the underlying mechanisms using tumor tissue. Western blot data showed that AZ505 single treatment could lead to the decrease of p53^K370me^, whereas CDDP single treatment could slightly increase the level of p53^K370me^ (Figure 5B). The combination treatment contributed to a decrease of p53^K370me^. In addition, we found the expression of the clv-PARP, an apoptosis biomarker, was increased in the combination group (Figure 5B). In summary, our *in vivo* data showed the inhibition of SMYD2 by AZ505 could sensitize cisplatin antitumor action through epigenetic regulation of p53.

## Discussion

Cisplatin(CDDP) is the first line drug for NSCLC patients, therefore, understanding and preventing CDDP resistance are considered as the crucial issue with respect to the treatment of NSCLC (5). Here, we found that SMYD2, a protein methyltransferase, was involved into cisplatin resistance. Furthermore, out data showed that SMYD2 expression and its activity were increasing in cisplatin resistant NSCLC cells. Mechanistically, SMYD2 prompt cell migration, increase the tumor sphere, and block apoptosis, which is dependent on the methylation of p53^K370^. The inhibition or knockdown of SMYD2 model would result in the increasing of sensitivity to cisplatin *in vitro* and *in vivo*. Our findings provide us with a novel perspective epigenetic regulation mechanisms underlying CDDP resistance and define that the combination of SMYD2 inhibitor and CDDP may have promise as treatments for patients with CDDP-resistant NSCLC.

SMYD2 is a protein methyltransferase that catalyzes the methylation of histone substrates, such as H3K4 and H3K36 (18), and non-histone substrates, including p53 (19), Rb (20), HSP90 (21), STAT3, and NF-κB (22). It has been reported that SMYD2 was involved into the upset and progression of various tumors, including leukemia, breast cancer, gastric cancer, and head and neck cancer. Recently, Wang et al. reported SMYD2 inhibition also led to the suppression of cell growth in NSCLC cells (23), suggesting SMYD2 might be involved into lung cancer. Our results demonstrated that SMYD2 expression and enzymatic activity levels were upregulated in NSCLC CDDP-resistant cells as compared to parental cells. In addition, either suppressing SMYD2 activity or knocking down SMYD2 would contribute to the increases in sensitivity to CDDP, and the reduction in cell migration and self-renewal ability in CDDP-resistant NSCLC cells, indicating that SMYD2 executes a crucial role in CDDP resistance of NSCLC.

SMYD2 methylates H3K4 and H3K36 would contribute the change of chromatin structure, and subsequently lead to the alteration of its target genes (18). In fact, the important function of SMYD2 was reported to related methylate to its non-histone substrates (9, 24). SMYD2 monomethylates Lys-370 of p53, leading to decreased DNA-binding activity and subsequent transcriptional regulation activity of p53. We found that, as long as the SMYD2 upregulation, the p53^K370me^ level was also increased in CDDP resistant NSCLC cells. Importantly, our data showed that cell sensitivity to CDDP was dependent on wild type p53 level. Inhibition of SMYD2 could induce the increasing of p53 transcription activity and its target gene expression. Taken together, these findings indicate that epigenetic regulation by SMYD2 plays an important role in p53 transcriptional activity and is involved in processes associated with CDDP resistance.

K-RAS is one of the most frequently mutated in human NSCLC (25). Mutation of K-RAS usually results in the activation of oncogenic signaling molecules that regulate cell growth, survival and differentiation by coupling receptor activation to downstream effector pathways (25), and leads to the resistance to tyrosine kinase inhibitors such as gefitinib and erlotinib (26). Therefore, chemotherapy is the standard of treatment for K-RAS mutant NSCLC tumors. Here, our data shown that inhibition of SMYD2 by specific inhibitor can sensitize CDDP efficacy in K-RAS mutated A549 and NCI-H460 cell lines, suggesting epigenetic manipulation might be a promising adjuvant approach to treat K-RAS mutant tumors.

In conclusion, the present study elucidated that the activity of SMYD2 in NSCLC may affect the cell sensitivity to chemotherapeutic agents, especially to CDDP. The elevated SMYD2 mediated CDDP resistance and malignant phenotype in NSCLC, indicating that SMYD2 may be a useful biomarker of CDDP resistance in NSCLC. Inhibition of SMYD2 contributes to the methylation-related activation of p53 and thus results in cell apoptosis. Furthermore, combination treatment with CDDP and an SMYD2 inhibitor had a synergistically antitumor effects in a xenograft model *in vivo*. Given that SMYD2 has reversible effects and is a targetable protein methyltransferase, treatments targeting the protein may be useful for reversing CDDP resistance in NSCLC.

## Ethics Statement

This study was carried out in accordance with the recommendations in the Guide for the Care and Use of Laboratory Animals of the National Institutes of Health, and the corresponding protocol was approved by the Animal Experimental Ethics Committee of Shenyang Medical College (Shenyang, Liaoning Province, China).

## Author Contributions

All authors listed have made a substantial, direct and intellectual contribution to the work, and approved it for publication.

### Conflict of Interest Statement

The authors declare that the research was conducted in the absence of any commercial or financial relationships that could be construed as a potential conflict of interest.

## Supplementary Material

Supplementary Figure 1 The effects of genetic or chemical manipulation of SMYD2 on the cell growth, migration, and tumor sphere ability of NCI-H460 cells. **(A)** The growth of A549 and NCI-H460 cells treated with various concentrations SMYD2 inhibitor BAY-598. Cell viability was detected by MTT assay. **(B)** The efficacy of genetic or chemical manipulation of SMYD2 was confirmed by Western blot in NCI-H460 cells. p53^K370me^ and SMYD2 expression levels were measured in NCI-H460 cell lines. The p53 or β-actin was used as a loading control. **(C)** Cell migration was measured in NCI-H460 cells treated with 2 μM BAY-598 or 50 nM SMYD2 siRNA. Scramble siRNA or DMSO was used as a control. **(D)** Tumor sphere was counted in NCI-H460 cells treated with 2 μM BAY-598 or 50 nM SMYD2 siRNA. Scramble siRNA or DMSO was used as a control.
