# Supplementary figures and images for: Inhibition of SMYD2 Sensitized Cisplatin to Resistant Cells in NSCLC Through Activating p53 Pathway
Source: Front Oncol. 2019 Apr 26;9:306. doi: 10.3389/fonc.2019.00306 (PMC6498871; doi:10.3389/fonc.2019.00306)

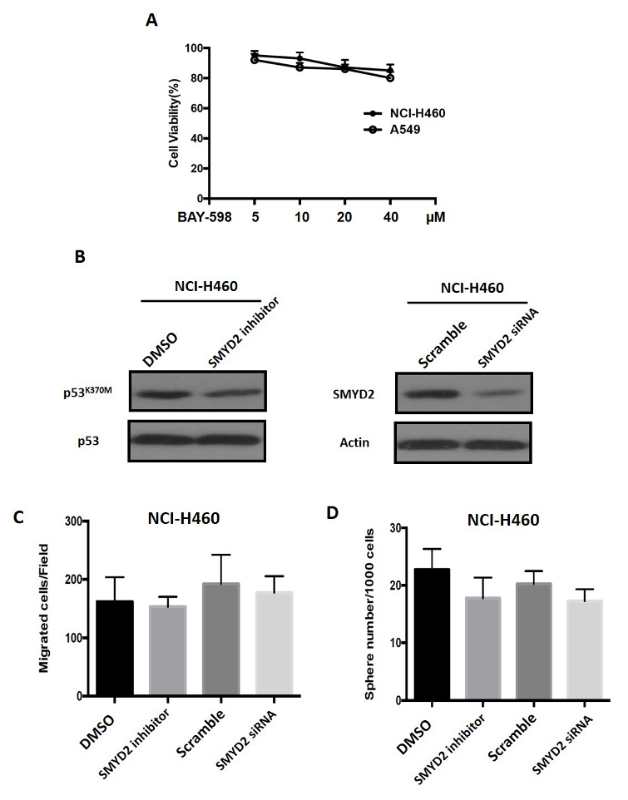

Supplement: Supplementary Figure 1 — The effects of genetic or chemical manipulation of SMYD2 on the cell growth, migration, and tumor sphere ability of NCI-H460 cells. (A) The growth of A549 and NCI-H460 cells treated with various concentrations SMYD2 inhibitor BAY-598. Cell viability was detected by MTT assay. (B) The efficacy of genetic or chemical manipulation of SMYD2 was confirmed by Western blot in NCI-H460 cells. p53K370me and SMYD2 expression levels were measured in NCI-H460 cell lines. The p53 or β-actin was used as a loading control. (C) Cell migration was measured in NCI-H460 cells treated with 2 μM BAY-598 or 50 nM SMYD2 siRNA. Scramble siRNA or DMSO was used as a control. (D) Tumor sphere was counted in NCI-H460 cells treated with 2 μM BAY-598 or 50 nM SMYD2 siRNA. Scramble siRNA or DMSO was used as a control. [file Image_1.JPEG]
